# Intensity interrogation near cutoff resonance for label-free cellular profiling

**DOI:** 10.1038/srep24685

**Published:** 2016-04-18

**Authors:** Yousef Nazirizadeh, Volker Behrends, Aurél Prósz, Norbert Orgovan, Robert Horvath, Ann M. Ferrie, Ye Fang, Christine Selhuber-Unkel, Martina Gerken

**Affiliations:** 1 Byosens GmbH, 20357 Hamburg, Germany; 2 Nanobiosensorics Group, Hungarian Academy of Sciences, Research Centre for Energy Research, Konkoly-Thege, 1120 Budapest, Hungary; 3 Biochemical Technologies, Science and Technology Division, Corning Incorporated, Corning, New York 14831, USA; 4 Institute for Materials Science, Christian-Albrechts-Universität zu Kiel, 24143 Kiel, Germany; 5 Institute of Electrical Engineering and Information Technology, Christian-Albrechts-Universität zu Kiel, 24143 Kiel, Germany

## Abstract

We report a method enabling intensity-based readout for label-free cellular assays, and realize a reader device with the same footprint as a microtiter plate. For unambiguous resonance intensity measurements in resonance waveguide grating (RWG) sensors, we propose to apply resonances near the substrate cutoff wavelength. This method was validated in bulk refractive index, surface bilayer and G protein-coupled receptor (GPCR) experiments. The significantly reduced size of the reader device opens new opportunities for easy integration into incubators or liquid handling systems.

For decades, labeling has been dominating most cellular assays for revealing microscopic processes within cells. Labels enable the researchers to address specific components of a complex mechanism in cells and allow applications ranging from intracellular imaging to detection of signal transduction pathways^1^. The label, however, has to be incorporated in the close vicinity of the mechanism of interest or must be directly expressed in cells through gene modification. Thus, labels might interfere with biological pathways or change the structure of very dense protein environments. Label-free methods, on the other hand, render tags, dyes and cell engineering unnecessary and enable highly sensitive pharmacological profiling in native cells^2^,^3^. One of the main label-free technologies applied in drug discovery is the resonance waveguide grating (RWG) biosensor, also known as photonic crystal sensor, which utilizes a surface-bound evanescent wave to probe the local refractive index at the sensor surface. This technology delivers an integrated and phenotypic response of whole cells in real time and can thus map cellular responses ranging from cell signaling to cell proliferation^4^,^5^. The groundbreaking potential of this technology is revealed when applying it to G protein-coupled receptor (GPCR) assays, since GPCRs represent the most addressed druggable targets in pharmaceutical research and development^6^. In contrast to traditional label-based GPCR assays, which mostly aim at a specific end-point in the signaling cascade, label-free technologies summarize all cellular events that occur during the stimulation^7^,^8^. The state-of-the-art readout of RWG sensors is through monitoring the shift in resonance wavelength or angle using sophisticated optics, electronics and mechanics^4^. Consequently, the integration of these complex and stationary instruments into the daily work process requires fundamental rearrangements. We here propose a novel, intensity-based readout for RWG sensors, permitting a mini reader with dimensions significantly smaller than the currently available instruments. The mini reader and the corresponding consumable, which can have microtiter plate format, can be easily placed in incubators and enable cellular assays in various ambient conditions. Furthermore, to manage fast cellular responses in a high-throughput manner, existing liquid handling systems can be utilized.

## Results

**Figure 1:**
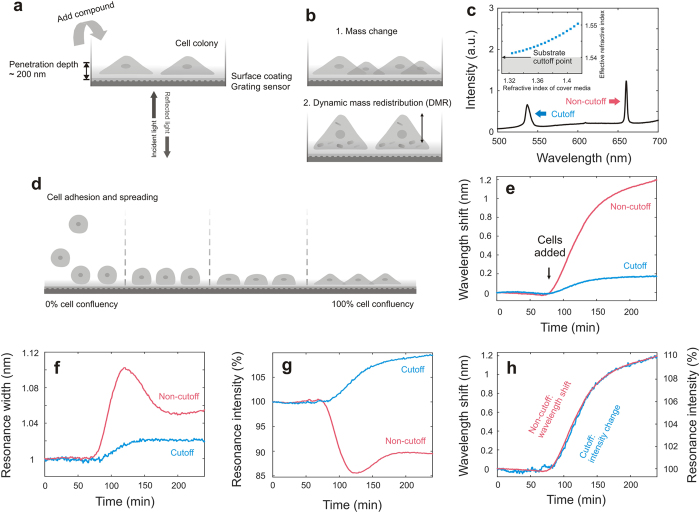
Resonant wavelength grating (RWG) sensor for cellular assays. (**a**) Illustration of the RWG sensor with a surface coating. The surface mass of cells grown on the surface is detected within the penetration depth. The interrogation of the RWG sensor in a spectral reflection measurement. (**b**) When compounds are added to the well, two main cellular responses are detected: (1) mass change and (2) dynamic mass redistribution (DMR). (**c**) Reflection spectrum of the RWG sensor under 26°. Blue and red arrows indicate the resonance at near-cutoff and non-cutoff wavelengths, respectively. The inset shows the appearing resonance mode curve close to the substrate cutoff for the TM_1_ mode. (**d**) Cell confluency in an adhesion and spreading assay. (**e**) Resonance central wavelength, (**f**) width change and (**g**) intensity change in percent during the adhesion and spreading assay. (**h**) The comparison of the central wavelength of the non-cutoff resonance and the intensity of the near-cutoff resonance shows that the intensity of the near-cutoff resonance can be utilized for surface mass detection.

A RWG sensor utilizes a periodically nanostructured waveguide to couple the incident light into the waveguide, and provide a leaky mode with an evanescent part, which is measured as a resonance in a reflection interrogation. The evanescent part of the mode typically exhibits a penetration depth of about 200 nanometers above the sensor structure (Fig. 1a), which is the sensing volume of the RWG sensor. Here we used Corning 96-well Epic microtiter plates with a RWG sensor integrated in each well.

During cell profiling two types of cell responses cause a refractive index change at the surface: (1) the increasing or decreasing number of cells on the sensor surface, and (2) the phenotypic response of cells that leads to the rearrangement of cellular mass, which is termed as dynamic mass redistribution (DMR) (Fig. 1b). The effective refractive index within the penetration depth and hence the surface mass correlates strongly to the resonance wavelength or angle. Tracking either the resonance wavelength or angle, the cellular surface mass can be obtained in real time^9^.

For biomolecular assays, a monotonous response is observed for both the resonance wavelength and the resonance intensity upon binding^10^. For cell confluency and GPCR experiments, there was a linear correlation between the resonance wavelength and surface mass, but an ambiguous relation between the resonance intensity and the surface mass^11^. The resonance intensity was not solely a function of the surface mass, but also a function of the change in scattering and cell confluency^12^. Hence, resonance intensity measurements previously were disregarded for measuring surface mass. We hypothesized that using a resonance near the substrate cutoff wavelength enables unambiguous resonance intensity measurements. The Corning RWG sensor permits two resonances: one close to the substrate cutoff wavelength (blue) corresponding to the first order transverse magnetic (TM_1_) mode and the other one far away from the cutoff wavelength (red) corresponding to the zero order transverse magnetic (TM_0_) mode (Fig. 1c). Theoretically investigating the mode characteristics by numerically solving the 3-layer mode equation^13^ suggests that the resonance near the cutoff point is disappearing when the refractive index of the cover medium is approaching down to 1.3 refractive index units (RIU) (inset in Fig. 1c). This is because the effective refractive index of the mode is approaching the refractive index of the substrate. At this point waveguiding is theoretically impossible and consequently the resonance peak disappears^14^,^15^. Therefore, near the cutoff point the resonance intensity must increase with increasing surface mass, giving a possibility for an alternative intensity-based interrogation with remarkable simplicity. Operating resonances near cutoff might also open interesting new directions for other detection principles, such as mechanical, electrical or magnetic sensing.

We validated this hypothesis in a cell adhesion experiment using a spectrometer. A Gaussian fit was applied to the reflected resonance to find its central wavelength, resonance width and peak value. The peak values were normalized to the peak value at the beginning of the experiment to obtain the percent of intensity change. Following the central wavelength, the cell confluency was increasing up to a maximum value due to cell adhesion and spreading (Fig. 1d)^16^. The adhesion and spreading of rat embryonic fibroblast (REF) cells increased the resonance wavelength until reaching a maximum for both resonances (Fig. 1e), but resulted in divergent behaviors in resonance width (Fig. 1f), or resonance intensity for both resonances (Fig. 1g). As the cells adhered and spread on the biosensor surface, the resonance width of the resonance at non-cutoff exhibited a local maximum (Fig. 1f) due to scattering losses reducing the quality factor of the biosensor, as well as due to the increased micron-scale inhomogeneity in surface mass^12^. As discussed in^11^,^17^ the resonance width is at its maximum, when the cell confluency reaches 50%. In Fig. 1f the local maximum is an indication for a cell confluency of 50%, which is exceeded during the adhesion process. However, the resonance nearby the substrate cutoff wavelength developed its full intensity with increasing surface mass, and its width showed a monotonous increase (Fig. 1f). The two resonances also displayed different kinetics in resonance intensity (Fig. 1g). In the non-cutoff resonance the intensity is a function of the quality factor and gave rise to a local minimum at cell confluency of 50%. In contrast to that, the intensity of the near-cutoff resonance followed the surface mass change monotonously. Almost identical kinetics were obtained for the cell adhesion process when the wavelength of the non-cutoff resonance and the intensity of the cutoff resonance were monitored (Fig. 1h).

**Figure 2:**
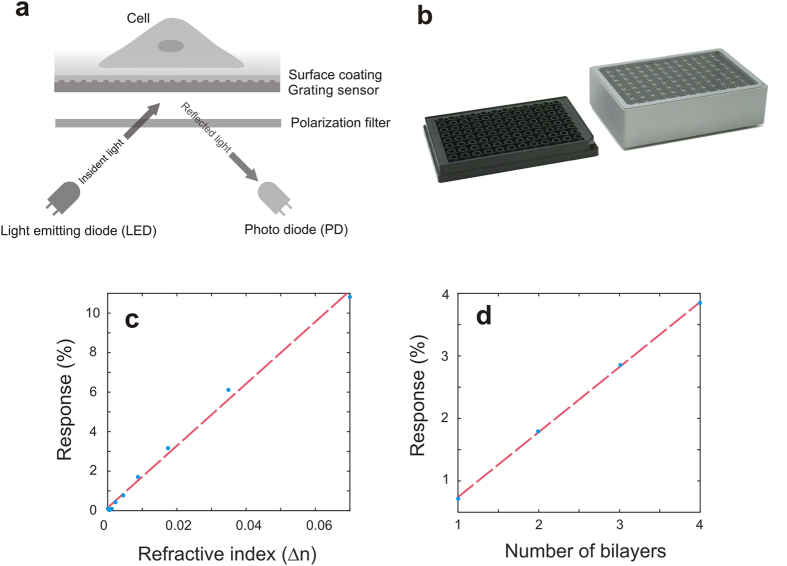
Intensity-based RWG sensor readout. (**a**) Schematic of the intensity-based readout consisting of a light emitting diode (LED) with collimation optics, photodiode with focusing optics and a circular polarization filter. (**b**) Demonstrator reader for 96-well microtiter plate (Epic from Corning). The footprint of this reader is the same as the microtiter plate itself. (**c**) Bulk refractive index experiments with glycerol dilutions in water. (**d**) Surface bilayer experiments with polyelectrolyte films.

By leveraging this finding, we designed a readout system using the near-cutoff resonance intensity without the need for a spectrometer and charge-coupled device. The readout components are reduced to a light emitting diode (LED) with a collimation optics, a photodiode with a focusing optics and a circular polarization filter to filter out parasitic reflections from the sensor downside (Fig. 2a). This component and size reduction enables a label-free microtiter plate reader with 96 readout units integrated onto a printed circuit board (PCB). The reader has the same footprint as the microtiter plate itself (Fig. 2b). The response of this reader to surface mass change was investigated in a bulk refractive index experiment with glycerol solutions, and in a surface layer-by-layer experiment with negatively charged poly(sodium 4-styrenesul- fonate) (PSS) and positively charged poly (allylamine hydrochloride) (PAH)^9^. The employed solutions were crosschecked with a refractometer. The first experiment, imitates a bulk mass change occurring in proliferation assays, while the second experiment imitates the surface mass change as found in DMR experiments. Both experiments showed linear responses to the mass changes (Fig. 2c,d, respectively).

**Figure 3:**
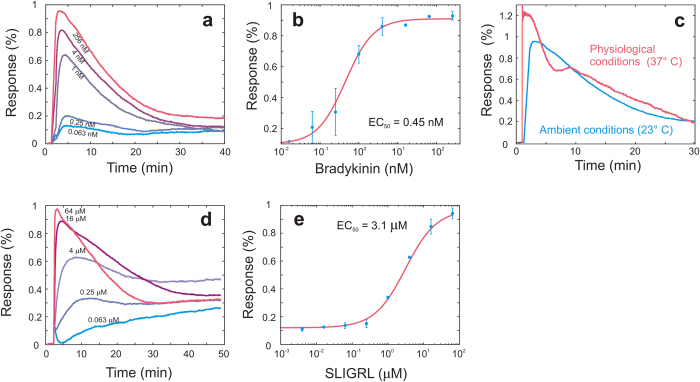
Validation experiments of the intensity based readout with G protein-coupled receptor (GPCR) assays. (**a**) Real-time response of A431 cells to bradykinin dose variations. (**b**) Dose response curve with duplicates of maximum response to bradykinin. The EC_50_ value derived from this curve is 0.45 nM. (**c**) Comparison of real-time response of A431 cells to bradykinin at ambient (23 °C) and physiological (37 °C) conditions. The maximum response and the curve shape show differences. (**d**) Whole cell real-time response of A431 cells to protease-activated receptor-2 activating peptide SLIGRL dose variations. (**e**) Dose response curve with duplicates of maximum response to SLIGRL resulting in an EC_50_ value of 3.1 μM.

The intensity-based interrogation method was also validated by GPCR experiments with bradykinin and protease-activated receptor-2 (PAR2) activating peptide SLIGRL. Bradykinin is an agonist for the bradykinin B2 receptor and activates both G_αq_ and G_αs_ pathways in A431 cells^18^. Results showed that bradykinin triggered a robust, dose-dependent intensity signal in A431 when assayed at ambient temperature (Fig. 3a), with a half maximal effective concentration (EC_50_) of 0.45 nM (Fig. 3b). The shape of the biosensor response curve, kinetics and EC_50_ obtained were all highly comparable to the results reported with a spectral interrogation reader^18^. Interestingly, compared to ambient conditions, bradykinin triggered an intensity signal having faster kinetics, greater amplitude and an additional shoulder when the cells were assayed under physiological conditions (37 °C) (Fig. 3c). This behavior was also observed in previous experiments^19^ and emphasized the importance of experiments under physiological conditions. The PAR2 agonist SLIGRL also triggered a robust, dose-dependent intensity signal in A431 cells (Fig. 3d), with an EC_50_ of 3.1 μM (Fig. 3e). Again, the shape, kinetics and EC_50_ obtained were all comparable to previous results measured with a spectral interrogation reader^20^.

In conclusion, we propose an intensity-based readout method for RWG sensors operating close to the substrate cutoff wavelength, and validated the functionality with bulk refractive index, surface bilayer and GPCR experiments. The described readout method enables reader instruments with a significantly smaller size. As the reader can have the same footprint as the microtiter plate, the integration of such a reader in existing equipments, e.g. incubators or liquid handling systems, is facilitated and can boost label-free detection usage. Moreover, this progress in form factor will possibly prompt and open label-free applications in fields other than pharmaceutical research and development, such as handheld devices for diagnostic applications^21^.

## Methods

### Optical setup for spectral measurements

For the optical investigation of the resonances, we used an in house built construction to address all 96 wells of the microtiter plate. It enables reflection measurements of angles from 5° to 50°. The light of a white LED was coupled to a multimode glass fiber and coupled out using a collimation optic directing to the RWG sensor. The reflected light was focused on a second glass fiber and coupled to a spectrometer. Every second, a spectrum was recorded and saved with a time stamp. During the measurement the wells of the microtiter plate were accessible and cells for the adhesion experiment could be added. In the experiment shown in Fig. 1, 25,000 REF cells were added to the uncoated well. After the experiment was performed both resonances were investigates using Matlab.

### Optical setup for intensity based interrogation

To measure the intensity of the resonance, we used a LED as the light source. Its peak wavelength was at 520 nm and was chosen to cover the spectral range of the resonance at 26°. An optics was used to collimate the LED light towards the RWG sensor, which had a spot size of about 1 mm. The reflected light was focused to a photo diode, which was driven in a short circuit mode with a subsequent transimpedance amplifier. The output voltage of the amplification was digitalized and normalized to the first value to obtain changes in percentage. Parasitic reflection, occurring from the edges of the well or liquid surface were suppressed using a circular polarization filter.

### Cells for adhesion experiments

Rat embryonic fibroblasts (REF52 wt) were maintained in Dulbecco’s Modified Eagle’s Medium (DMEM, Biochrom) supplemented with 10% FBS (Biochrom) and Penicillin-Streptomycin (working concentration: 50 U/ml Penicillin, 50 μg/ml Streptomycin, Biochrom) at 37 °C, 5% CO_2_ and about 90% humidity. Cells were trypsinized (0.25% trypsin/0.05% EDTA, Biochrom) and added to the Epic 96 well biosensor plates.

### Bulk refractive index experiments

99.5% glycerol from Sigma was dissolved in Milli-Q (MQ) water in various concentrations. A Rudolph J157 table top refractometer (Rudolph Research Analytical, Hackettstown, NJ, USA) was used to determine the refractive index values of the employed glycerol solutions. The refractometer had a precision of 10^−5^ RIU. First, 50 μl of pure water was pipetted into the wells to establish a baseline. Subsequently, the water was removed and glycerol solutions in the order of increasing concentration were added with a pipette to the well. The sensor signals were recorded for 5 minutes for each concentration. No washing with MQ water was performed between the applications of the various solutions; the solutions were removed by taking the plate out from the instrument and shaking the solutions from the wells. During this process, the measurement was always paused.

### Surface bilayer experiments

For the layer-by-layer assembly, we used negatively charged poly(sodium 4-styrenesulfonate) (PSS) with an average molecular weight of 70 kDa (Sigma-Aldrich Chemie GmbH, Munich, Germany) and positively charged poly(allylamine hydrochloride) (PAH) with an average molecular weight of 160 kDa (Alfa Aesar GmbH, Karlsruhe, Germany). PAH and PSS were dissolved in 10 mM 4-(2-hydroxyethyl)-1-piperazineethanesulfonic acid (HEPES) buffer (pH 7.4 was set with KOH) to a final concentration of 1 mg/ml. First, 50 μl of pure buffer was pipetted into the wells to establish a baseline. Afterwards, 50 μl of PSS and PAH were added to the wells in an alternating manner, performing three washing steps with pure buffer before each polyelectrolyte deposition. The exchange of solutions and the recording of the signals were carried out as in the previous experiment.

### Cells for GPCR experiments

Human epidermoid carcinoma A431 cell line was obtained from American Type Cell Culture (Manassas, VA, USA). A431 cells were passaged at 37 °C with 5% CO_2_ using Dulbecco’s Modified Eagle’s Medium (DMEM) supplemented with 10% fatal bovine serum (FBS), 4.5 g/L glucose, 2 mM glutamine, 100 μg/ml penicillin and streptomycin. The cells were passed with trypsin/ethylene-diaminetetraacetic acid when approaching 90% confluence to provide new maintenance culture on T-75 flasks and experimental culture on the biosensor microplates. For cell profiling, 100,000 cells in the serum-containing medium were seeded into each well of Epic 96-well biosensor plates, and cultured overnight. The cells were then washed three times and starved with the serum free medium for ~24 hr. Finally, the cells were washed using the HBSS buffer (1x Hanks’ balanced salt buffer, 10 mM HEPES-KOH, pH 7.1) before assay.

## Additional Information

**How to cite this article**: Nazirizadeh, Y. *et al.* Intensity interrogation near cutoff resonance for label-free cellular profiling. *Sci. Rep.* **6**, 24685; doi: 10.1038/srep24685 (2016).
